# Complexation between the Antioxidant Pterostilbene and Derivatized Cyclodextrins in the Solid State and in Aqueous Solution

**DOI:** 10.3390/ph16020247

**Published:** 2023-02-07

**Authors:** Laura Catenacci, Alexios I. Vicatos, Milena Sorrenti, Cesarina Edmonds-Smith, Maria Cristina Bonferoni, Mino R. Caira

**Affiliations:** 1 Department of Drug Sciences, University of Pavia, Viale Taramelli 12, 27100 Pavia, Italy; 2 Department of Chemistry, University of Cape Town, Rondebosch 7701, South Africa

**Keywords:** pterostilbene, derivatized cyclodextrins, inclusion complexes, thermal analysis, Fourier transform infrared spectroscopy, phase solubility studies

## Abstract

Inadequate aqueous solubilities of bioactive compounds hinder their ability to be developed for medicinal applications. The potent antioxidant pterostilbene (PTB) is a case in point. The aim of this study was to use a series of modified water-soluble cyclodextrins (CDs), namely, hydroxypropyl β-CD (HPβCD), dimethylated β-CD (DIMEB), randomly methylated β-CD (RAMEB), and sulfobutyl ether β-CD sodium salt (SBECD) to prepare inclusion complexes of PTB via various solid, semi-solid, and solution-based treatments. Putative CD–PTB products generated by solid-state co-grinding, kneading, irradiation with microwaves, and the evaporative treatment of CD–PTB solutions were considered to have potential for future applications. Primary analytical methods for examining CD–PTB products included differential scanning calorimetry and Fourier transform infrared spectroscopy to detect the occurrence of binary complex formation. Phase solubility analysis was used to probe CD–PTB complexation in an aqueous solution. Complexation was evident in both the solid-state and in solution. Complex association constants (K_1:1_) in an aqueous solution spanned the approximate range of 15,000 to 55,000 M^−1^; the values increased with the CDs in the order HPβCD < DIMEB < RAMEB < SBECD. Significant PTB solubility enhancement factors were recorded at 100 mM CD concentrations, the most accurately determined values being in the range 700-fold to 1250-fold.

## 1. Introduction

In recent years, the number of research articles on the pharmacological activity of the natural polyphenol pterostilbene (3,5-dimethoxy-4’-hydroxystilbene, PTB), belonging to the stilbene class, has significantly increased, confirming its potential use for the treatment of various human diseases. PTB is well-known for its anti-inflammatory [1,2], antioxidant [3], anticancer [4,5], and neuroprotective activity and it shows promise for the treatment of complications of SARS-CoV-2 [6,7].

Although the presence in the PTB structure of two methoxy groups contributes to its higher lipophilicity compared to that of its analogue resveratrol (3,4’,5-trihydroxystilbene), its use in biomedical applications is hampered due to its poor water solubility and consequent low bioavailability.

In our previous paper [8], we investigated the feasibility of including PTB in native cyclodextrins (CDs) to increase its solubility, presenting results that showed significant solubility enhancements using α-, β-, and γ-CD and deriving the respective complex association constants from phase solubility data. The application of CD inclusion complexes represents, in fact, one of the most useful approaches to enhance the solubility of compounds belonging to class II of the Biopharmaceutical Classification System (BCS), other methods including the reduction in particle size to increase the surface area [9], nanotechnology [10], and salt formation [11].

In the study reported here, we investigated the feasibility of complexation between PTB and several pharmaceutically relevant derivatives of β-CD, specifically, hydroxypropyl-β-CD, (HPβCD), heptakis(2,6-di-*O*-methyl)-β-CD (DIMEB), randomly methylated β-CD (RAMEB), and sulfobutyl ether β-CD sodium salt (SBECD). The host compound heptakis(2,3,6-tri-*O*-methyl) β-CD (TRIMEB) was also used in an attempt to obtain a complex for possible structural analysis by X-ray methods.

## 2. Results

The study of the complexation of PTB with derivatized CDs using different preparative techniques required a preliminary characterization of PTB itself with the same techniques used for the preparation of the binary CD–PTB systems.

Thermal techniques [thermogravimetric analysis (TGA) and differential scanning calorimetry (DSC)] indicated that the commercial PTB used in this study melts at 95.4 ± 0.2 °C (T_onset_ = 94.6 ± 0.1 °C; ΔH_m_ = 91 ± 1 J g^−1^) and subsequently decomposes at approximately 220 °C, confirming that this phase corresponds to the stable monoclinic polymorph I described in the literature [12].

In Table 1, we report the resulting thermal parameters of the solid phases obtained by the treatment of commercially available PTB with various preparative techniques to verify that no significant changes in its solid state and/or its physical stability occurred.

The derived products were obtained by solution-based, semi-solid, and solid techniques including solid-state grinding (GR), microwave irradiation (MW), kneading (KN), and the treatment of PTB solutions with a rotavapor apparatus (RV). The data in Table 1 show that a significant reduction in the melting enthalpy value was recorded for the MW product only, indicating a partial amorphization of the PTB resulting from this preparative method. For all the other samples, the data confirm that the product isolated corresponded to the commercial material employed, namely, the same, more stable PTB polymorphic form I.

### 2.1. Isolation and Characterization of Solid Complexes between Derivatized CDs and PTB

The thermal parameters of the commercial PTB were used as references for the pure compound in the study of the interaction products. The various methods listed above were employed in the preparation of PTB inclusion complexes with the selected derivatized β-CDs. Physical mixtures (PMs) with each CD were also prepared.

#### 2.1.1. PTB + HPβCD

**Figure 1:**
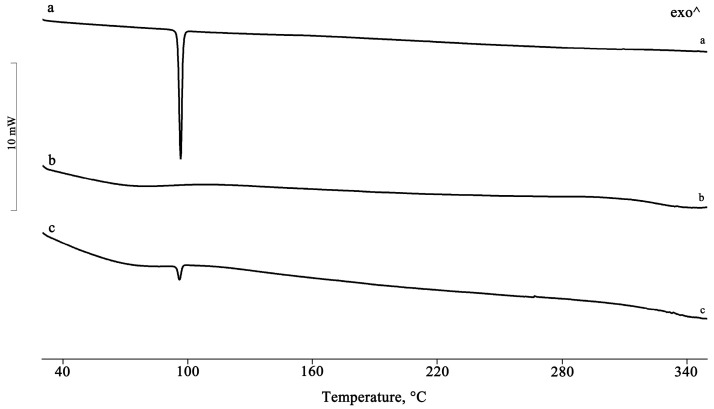
DSC profiles of the commercial PTB (**a**), HPβCD (**b**), and their PM (**c**).

Figure 1 shows the DSC curves of PTB, HPβCD, and their equimolar PM.

**Figure 2:**
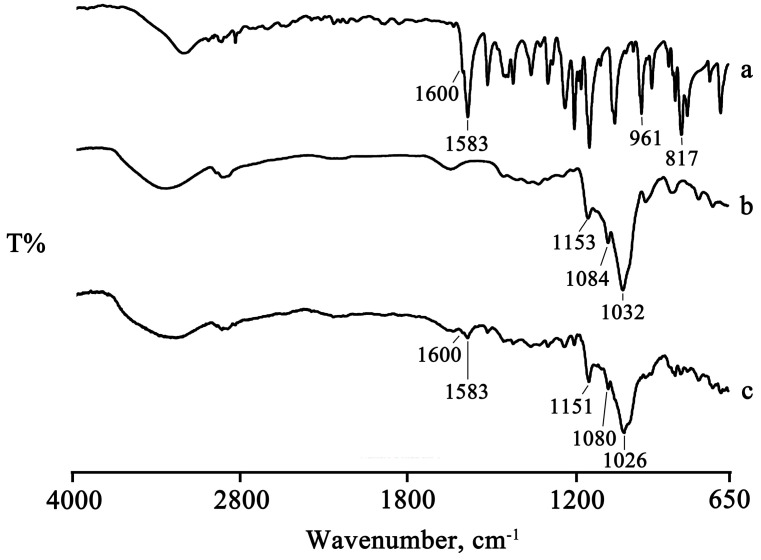
FTIR spectra of the commercial PTB (**a**), HPβCD (**b**), and their PM (**c**).

The presence of the drug melting endotherm of PTB is still evident at 95.5 ± 0.5 °C in the PM, with a reduction in the melting enthalpy value by 20% relative to that of the pure PTB, probably due to a partial interaction and/or amorphization of the system. This partial interaction was confirmed by FTIR analysis recorded on the same samples, for which it was possible to note a shift of the characteristic absorption bands of the CD at 1153, 1084, and 1032 cm^−1^ toward slightly lower wavenumbers, namely, 1151, 1080, and 1026 cm^−1^, respectively (Figure 2).

**Figure 3:**
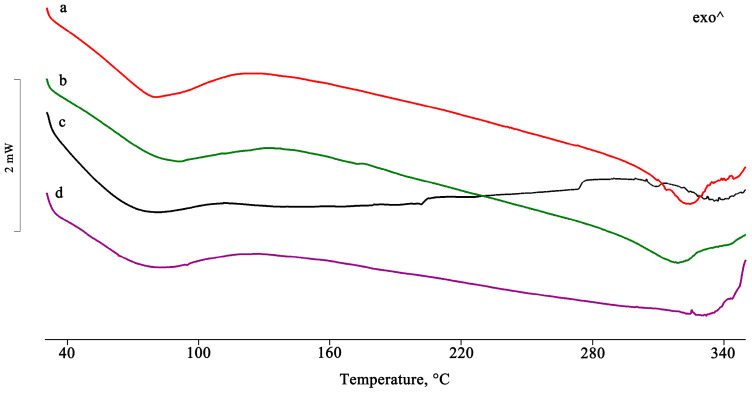
DSC profiles of the PTB + HPβCD PM treated by the GR, MW, KN, and RV methods (**a**–**d**).

For the samples isolated using the other preparative methods, the complete disappearance of the PTB melting peak indicates the inclusion of the active compound in the hydrophobic CD cavity (Figure 3).

**Figure 4:**
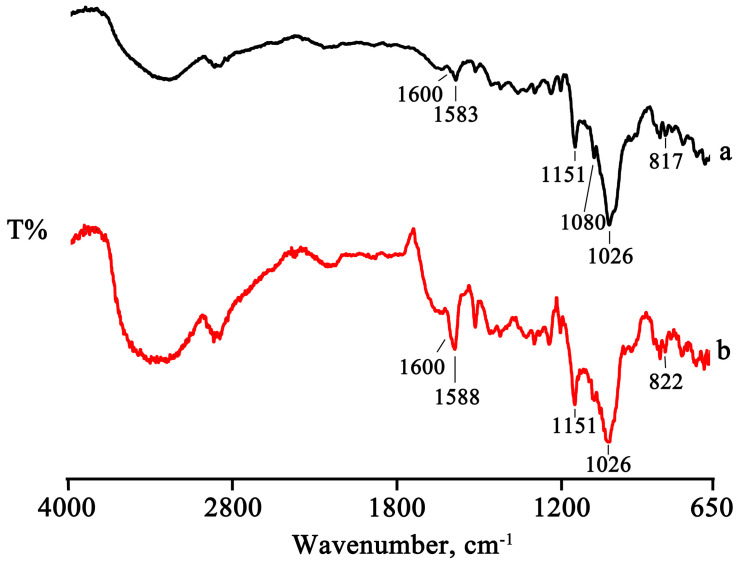
FTIR spectra of PTB + HPβCD PM (**a**) and GR product (**b**).

The thermal data are supported by the FTIR analysis. The two characteristic bands of PTB at 1583 cm^−1^ (related to the stretching of olefinic C = C) and at 817 cm^−1^ (related to the vibrations of the C–H groups), which are still present in the PM spectrum at the same wavenumbers, shifted to 1588 cm^−1^ and 822 cm^−1^, respectively, in the spectra of the treated products, as can be seen in the spectrum of the GR product as representative, as shown in Figure 4.

#### 2.1.2. PTB + DIMEB

**Figure 5:**
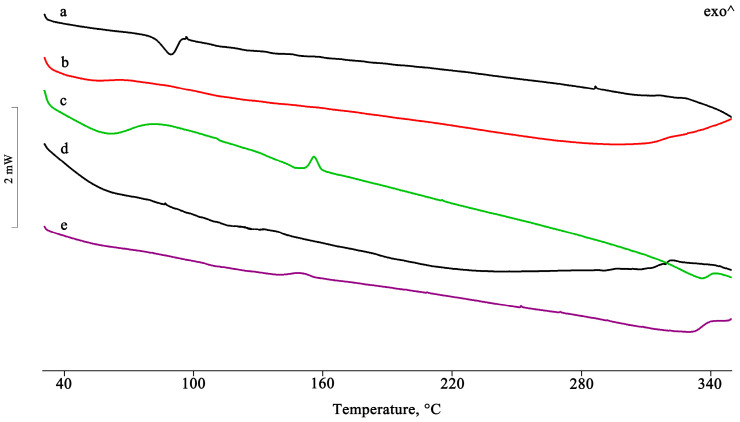
DSC profiles of PTB + DIMEB PM as such (**a**) and after treatment by GR, MW, KN, and RV (**b**–**e**).

In the PTB-DIMEB PM, the melting enthalpy value of PTB form I was much lower than that of the pure material (Figure 5), thus indicating that with DIMEB, a relatively strong host–guest interaction results via simple mixing of the two components. In contrast, the melting endotherm of PTB disappeared in the other treated samples, indicating a complex formation. Moreover, in the binary systems obtained from MW and RV, an endo/exothermic effect appeared in their DSC traces at 155 and 140 °C, respectively, confirming the inclusion of PTB in the cavity of DIMEB.

**Figure 6:**
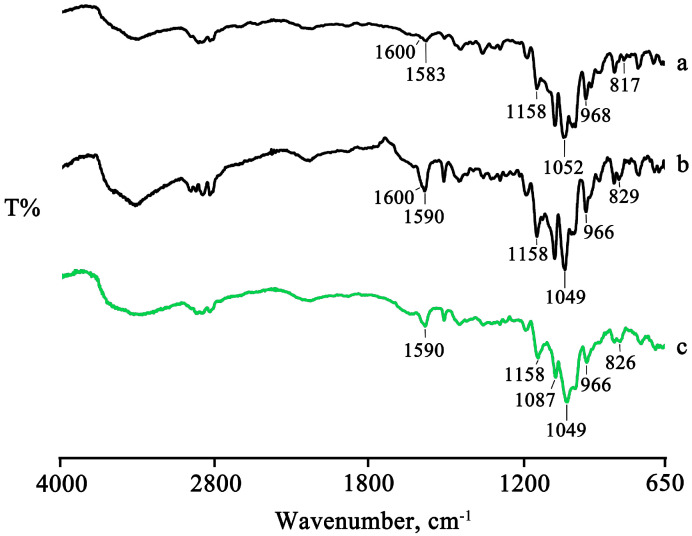
FTIR spectra of PTB + DIMEB PM (**a**), KN (**b**), and MW (**c**) products.

The spectra of the treated products showed a shift to slightly higher wavenumbers or the disappearance of some characteristic bands of PTB as a result of the host–guest interactions (Figure 6), supporting the interpretation above based on the thermal data.

**Figure 7:**
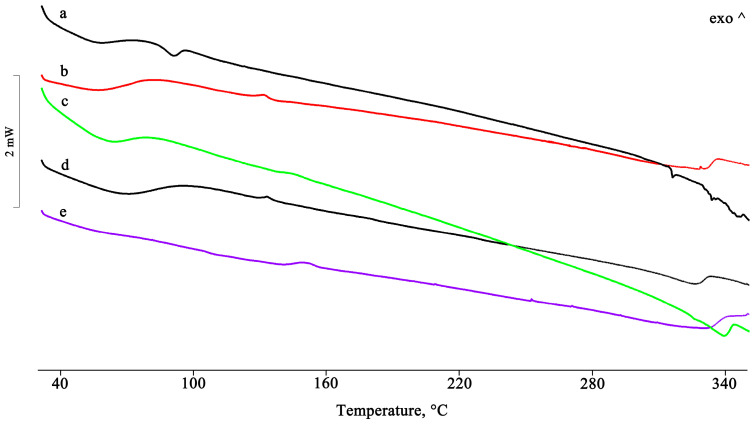
DSC profiles of the PTB + DIMEB 1:2 (mol/mol) PM (**a**) and the GR, KN, RV, and MW products (**b**–**e**).

For the dimethylated derivative (DIMEB), in addition to its equimolar PM with PTB, a PTB + DIMEB 1:2 mol/mol system was also prepared by physical mixing (i.e., a binary system containing an excess of the CD). For this different stoichiometric ratio, it was possible to draw the same conclusions as for the equimolar system. In the DSC profiles of the 1:2 mol/mol PM, the melting peak of PTB was still present (Figure 7), albeit with a reduced enthalpy compared to that of the commercial material, but it disappeared in the treated products as a consequence of the host–guest complex formation. The interaction between the two components was also supported by the appearance of an endo/exothermic effect at about 140 °C for all systems treated, as recorded for the equimolar system only from the MW and RV products.

For this binary system, hot stage microscopy (HSM) analysis was carried out with the samples immersed in silicone oil to facilitate the identification of the thermal effects.

**Figure 8:**
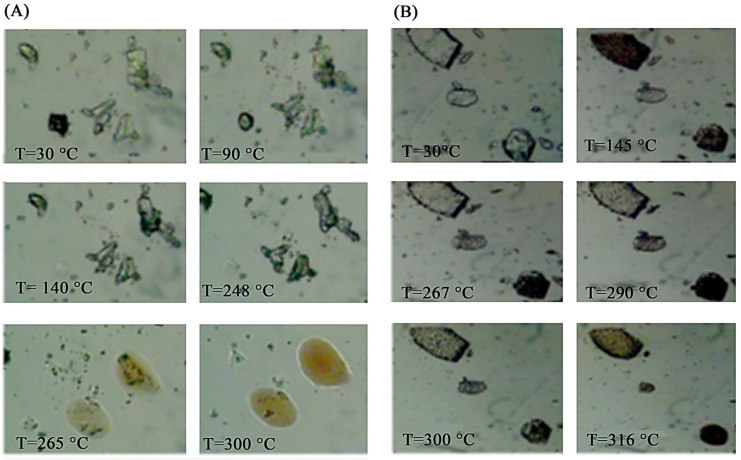
HSM photomicrographs of the PTB + DIMEB 1:2 (mol/mol) PM (**A**) and that recorded for the RV product (**B**) at the various temperatures reported in the images.

The photomicrograph recorded for the PM (Figure 8A) showed two distinct crystal phases, namely, the platy one of PTB and the columnar one of DIMEB, which melted at the characteristic temperatures of the two single components (viz. ≈90 °C for PTB and ≈240 °C for DIMEB); the RV sample, as a representative case of a treated product, showed a different crystal morphology confirming the isolation of a new phase (Figure 8B). During heating, a browning of some crystals was observed at the same temperature at which the endo-exothermic effect in DSC was recorded (≈145 °C), followed by a brightening (from ≈260 °C to ≈290 °C) until the sample completely decomposed at about 300 °C.

The PTB-DIMEB interaction in the product with 1:2 stoichiometry is supported, as before, by the FTIR spectra, where a shift and/or a disappearance of some typical bands of PTB is evident (data not shown).

#### 2.1.3. PTB + RAMEB

**Figure 9:**
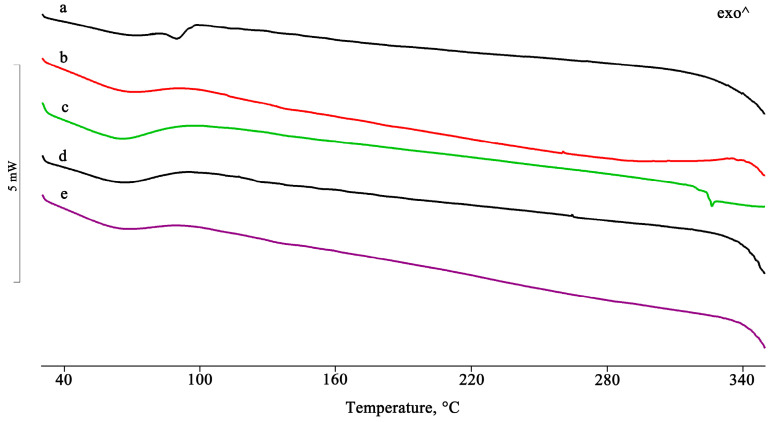
DSC profiles of PTB + RAMEB PM (**a**) and the GR, KN, RV, and MW products (**b**–**e**).

**Figure 10:**
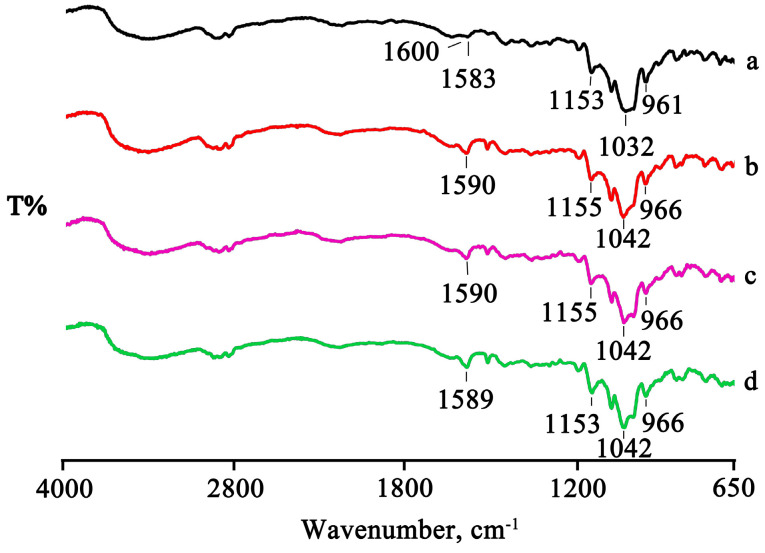
FTIR spectra of the PTB + RAMEB PM (**a**) and the GR, RV, and MW products (**b**–**d**).

For the binary system obtained with randomly methylated β-CD (RAMEB), the thermal and spectroscopic data were very similar to those obtained using the dimethylated derivative. Additionally, for this system, an interaction was already evident following simple mixing, both from the thermal analysis (disappearance of the PTB melting peak, Figure 9) and the spectroscopic analysis (shift and/or disappearance of absorption bands, Figure 10). This interaction reached completion in the treated systems.

#### 2.1.4. PTB + TMB

**Figure 11:**
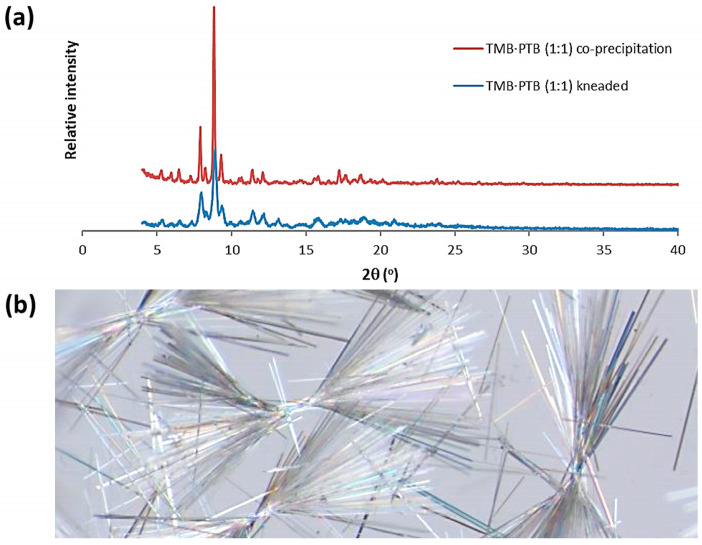
PXRD patterns of the 1:1 TMB·PTB complex prepared by kneading and co-precipitation (**a**), and the morphology of the product obtained by co-precipitation (**b**).

While the modified CDs referred to in this study have very significant advantages for drug delivery, their solid inclusion complexes containing bioactive guest molecules are generally amorphous, which severely limits the extent of the structural information on the host–guest interactions that can be derived by physical methods. In an attempt to obtain such information for an inclusion complex between a modified CD and PTB, we selected permethylated β-CD (TMB, for ‘trimethylated β-CD’) as a potential host, given the crystalline nature of both this host and its inclusion complexes, and hence also given the possibility of using X-ray methods of analysis for structure determination. Kneading of an equimolar mixture of TMB and PTB with water as the medium yielded a crystalline product, as indicated by PXRD (Figure 11a).

Complexation between TMB and PTB via co-precipitation in an aqueous solution was found to occur optimally when a 2:1 host–guest ratio was used. However, ^1^H NMR analysis of the filtered crystalline product dissolved in deuterated dimethyl sulfoxide (DMSO-d_6_) at 23 °C revealed that its host–guest stoichiometry was 1:1 (Supplementary Materials, Figure S1: The structures of PTB and TMB and the ^1^H NMR spectrum of the inclusion complex TMB·PTB).

**Figure 12:**
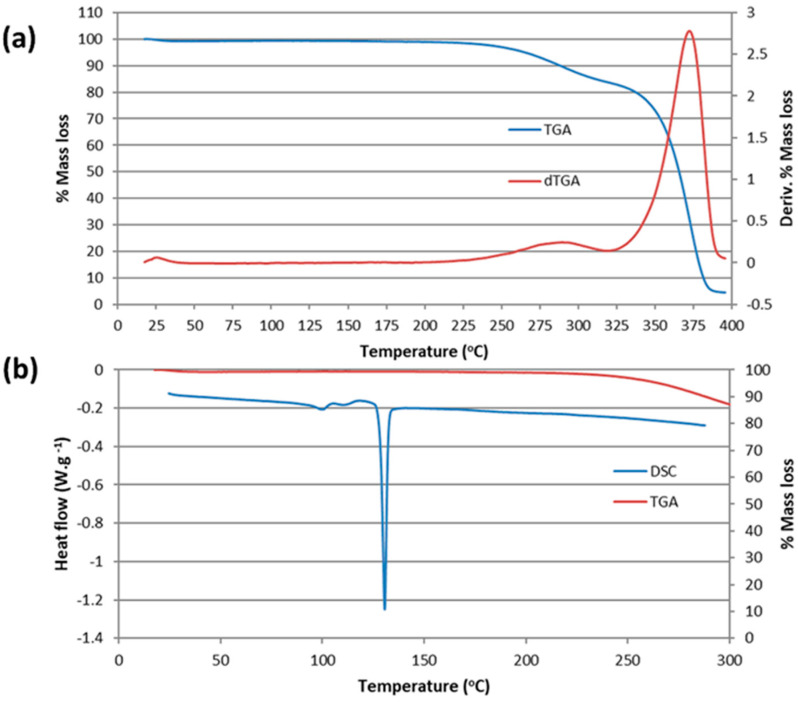
The TGA/dTGA curves (**a**) and the DSC curve with the overlaid TGA curve for reference (**b**) for the TMB·PTB complex.

The acicular crystals of the TMB·PTB complex associate in ‘bowtie’ motifs (Figure 11b), with the average thickness of a single crystal whisker being only 0.04 mm. Single-crystal X-ray diffraction (SCXRD) analysis was attempted, but the crystals were generally twinned and diffracted poorly due to their small volume, and unfortunately, a full data-collection could not be achieved. However, one specimen yielded an orthorhombic unit cell with the dimensions a = 14.737(3) Å, b = 30.886(7) Å, c = 58.77(2) Å, V = 26.750(20) Å^3^, which did not, however, coincide with those of any known TMB complex structure [13]. Despite the lack of a crystal structure, detailed analytical characterization of this new crystalline phase was nevertheless desirable. The HSM of a single crystal (not shown) indicated a melting onset at 95 °C and complete melting at 133 °C, while sublimation was evident in the temperature range 207–244 °C. Figure 12 shows the results of the TGA and DSC analyses of the complex. A small dehydration step with a mass loss 0.6 ± 0.2% occurred in the temperature range 21.5 ± 2.7 °C and 36.6 ± 0.6 °C, which equated to 0.6 ± 0.1 water molecules per TMB·PTB complex unit. Expulsion of the guest commenced at 166.1 ± 7.2 °C (mass loss 14.9 ± 1.4%, vs. the theoretical value of 15.2% for 1:1 host–guest stoichiometry) and was followed by sublimation of the host. The formula of the complex, TMB·PTB·0.6 (H_2_O), was thus confirmed by a combination of ^1^H NMR spectroscopic and TGA data.

Finally, the DSC curve revealed two small endotherms that were interpreted as possible phase transitions that preceded the sharp complex melting event at 130.6 ± 0.1 °C, this temperature being consistent with the HSM estimate of 133 °C.

### 2.2. Phase-Solubility Analyses

**Figure 13:**
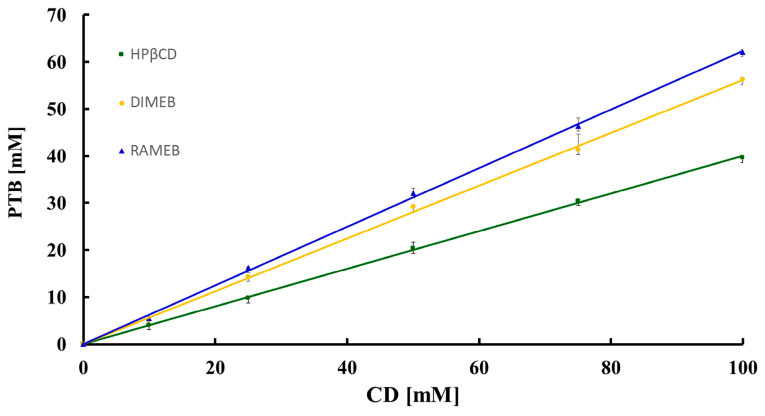
Phase-solubility profiles of PTB as a function of [HPβCD] (green), [DIMEB] (yellow), and [RAMEB] (blue).

In Figure 13, the phase-solubility profiles of PTB as a function of the HPβCD, DIMEB, and RAMEB concentrations are shown.

All resulting profiles were of type A_L_ [14] with an increase in the apparent solubility of PTB (for which the inherent aqueous solubility *S*_0_, determined under the same experimental conditions, is 54 ± 7 μM at 23 °C) due to soluble complex formation. Specifically, the solubility of the active compound, at the maximum CD concentration (100 mM), attained the values of 41.9 ± 0.3 mM, 57.6 ± 0.1 mM, and 60.4 ± 0.4 mM with HPβCD, DIMEB and RAMEB, respectively, these values corresponding to a 700-fold, 1200-fold, and 1250-fold increase over the solubility *S*_0_.

The apparent stability constant *K*_1:1_ of the complexes was estimated using the equation:

$$K_{1:1}=\frac{Slope}{S_{0}\left(1-Slope\right)}$$

where the slope was calculated from the linear phase-diagram profiles. The 1:1 apparent stability constants of the complexes formed with HPβCD, DIMEB, and RAMEB were 14,889, 25,118, and 30,104 M^−1^, respectively, indicating relatively strong interactions between PTB and these derivatized CDs. For the same systems, the complexation efficiency (CE) was evaluated using the equation:

$$CE=S_{0}xK_{1:1}=\frac{\left[D/CD\right]}{\left[CD\right]}=\frac{Slope}{\left(1-Slope\right)}$$

The CE values obtained were 0.7, 1.2, and 1.4 for the PTB-HPβCD, PTB-DIMEB, and PTB-RAMEB systems, respectively, indicating an improved efficiency of complexation effected by DIMEB and RAMEB.

**Figure 14:**
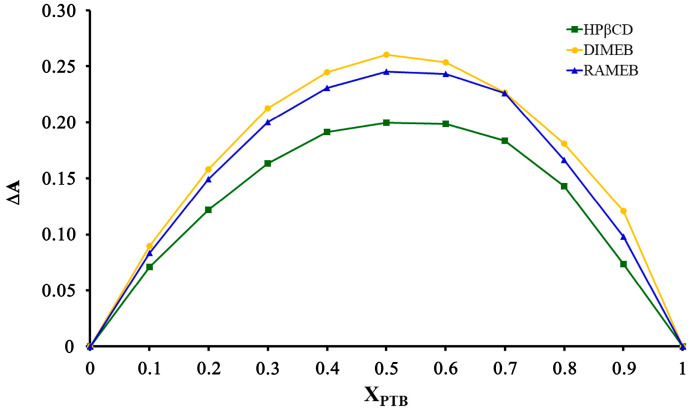
Job plots for the PTB-derivatized CD complexes.

To confirm the stoichiometries of the inclusion complexes, Job’s method was carried out for the three systems. The results obtained (Figure 14) indicate that for all three systems, the curve maxima corresponded to X_PTB_ ≈ 0.5, confirming the equimolar host–guest stoichiometries.

To complement the phase solubility study reported above using HPβCD, an analogous experiment with the modified cyclodextrin sulfobutyl ether β-CD sodium salt (SBECD) was performed to investigate its solubilizing power for PTB. Both HPβCD and SBECD are potent solubilizers with aqueous solubilities exceeding 1200 mg/mL. Furthermore, they are exceptional in that both have been approved not only for general routes of administration, but also for parenteral applications [15].

The phase solubility profile of PTB as a function of SBECD concentration was determined over the range 0–20 mM due to the limited availability of the host compound (Supplementary Materials, Figure S2: HPLC calibration curve and phase-solubility diagram). Using the same procedure as that used for the previous CDs, this profile can be assigned as the A_P_-type since the trendline displayed an overall slightly positive exponential tendency. Based on the value of the linear slope between 0 and 4 mM, the calculated K_1:1_ value was ≈55,200 M^−1^, indicating very strong host–guest binding and the complexation efficiency (CE) was 3.0. The PTB solubility enhancement factor at a SBECD concentration of 20 mM was ≈500-fold, but for comparison with the enhancement factors listed above for HPβCD, DIMEB, and RAMEB at concentrations of 100 mM, extrapolation of the trendline for SBECD to 100 mM reflected an estimated solubility enhancement factor of ≈5000-fold.

## 3. Discussion

The data listed in Table 1 clearly indicate that the polymorphic form of PTB used in this study corresponds to the commercially available form I and that this form is maintained following processing by the various tabulated methods. This polymorphic identification, which we also established previously by powder X-ray diffraction (PXRD) [8], is significant as PTB is known to exist in at least four polymorphic modifications [16], and attempted complexation with a given CD using solid- and semi-solid preparative techniques could conceivably yield different results, depending on which polymorph of PTB is employed.

Formation of solid inclusion complexes between PTB and the modified CDs HPβCD, DIMEB, and RAMEB via the GR, MW, KN, and RV preparative methods was conveniently determined using the combined techniques of DSC and FTIR spectroscopy. On examining the products, the thermal effects gleaned from the former technique included a significant reduction in the PTB melting enthalpy or disappearance of the PTB melting peak as well as significant endo/exothermic events, thus generally indicating host–guest interaction, while the FTIR spectra supported interpretations from the DSC curves by revealing significant shifts in the diagnostic absorption peaks of PTB. Evidence for the formation of two distinct complexes between DIMEB and PTB with 1:1 and 1:2 host–guest stoichiometries was obtained using HSM, which revealed both morphological differences between the products and differences in their thermal behaviors.

Thus, one of the aims of the study was fulfilled and the successfully isolated solid complexes identified will undergo further development including the solubility determination for possible improvements in PTB solubility and stability testing under different temperature and humidity conditions.

We recently reported the X-ray crystal structure of a β-CD·PTB inclusion complex [8] which showed, for the first time, the mode of inclusion of a PTB molecule within the cavity of a CD molecule. In an effort to obtain analogous structural information from a PTB complex with a modified CD, we chose the fully methylated, crystalline host compound TMB. As described above, isolation of a 1:1 complex was achieved by both kneading and co-precipitation methods and was analyzed by ^1^H NMR spectroscopy and the HSM, TGA, and DSC methods, which together indicated the complex stoichiometry, melting behavior, and crystal water content, and revealed the onset of mass loss of the PTB molecule upon heating the complex to ~166 °C. The expectation of determining the definitive structural features of the TMB·PTB complex for comparison with those we established earlier for the β-CD·PTB complex using SCXRD was not met, however, due to the intractable nature of the crystals. Although interesting structural differences in the modes of inclusion for a common guest in β-CD and methylated CDs may occur, the desired outcome in the present case would, however, have been one of academic interest rather than applicability, given the unsuitability of TMB as a vehicle for parenteral drug delivery due to its hemolytic effects and tendency to sequester cholesterol from membranes [17]. Due to these shortcomings, there was also no merit in proceeding with phase solubility analysis with TMB as the host compound.

The second aim of the study, namely, the acquisition of new data for PTB complexation with four pharmaceutically relevant modified CDs in aqueous solution, was likewise fulfilled. These data included the estimated complex association constants (K_1:1_), complexation efficiency (CE) values, and solubility enhancement factors for PTB resulting from its complexation with the series of derivatized CDs in an aqueous medium. The values of K_1:1_, namely, 14,889, 25,118, 30,104, and ~55,200 M^−1^ spanned a wide range, reflecting the increasing strengths of PTB binding to the respective CDs in the order HPβCD < DIMEB < RAMEB < SBECD. A value of K_1:1_ for HPβCD–PTB interaction was reported earlier as 17,520 M^−1^ at pH 7.0 [18] from a study based on measuring changes in the fluorescence spectroscopic properties of PTB in the presence of HPβCD. Despite the different methods employed, this value is in fair agreement with the value of 14,889 M^−1^ at pH 7.4 obtained in the present study. Given that the macrocyclic ring size is constant for the CD series employed in this study, it is evident that the PTB-CD binding strengths depend critically on the structural and chemical nature of the respective substituents attached to the oxygen atoms, and that (e.g., the CDs with methyl groups in closer proximity to their respective cavities, *viz.* DIMEB, RAMEB) promote stronger host–guest binding than HPβCD. Impressive PTB solubility enhancement factors in the range 700 to 1250 times that of pure PTB were attained as a result of its dissolution in 100 mM solutions of HPβCD, DIMEB, and RAMEB. As expected, the range of K_1:1_ values obtained with the derivatized CDs far exceeded the range reported previously for the interactions of PTB with α-, β-, and γ-CD, viz. 133 M^−1^ to 4950 M^−1^ [8].

From the phase solubility study of PTB with the polyanionic CD derivative SBECD, an unusually high value of the association constant K_1:1_ (≈5 × 10^4^ M^−1^) was obtained, implying very strong host–guest binding and a very close fit of PTB within the cavity of the CD molecule. This feature might render its release for delivery unfavorable. However, as SBECD can be administered intravenously, injection of a small volume of its PTB solution could increase the PTB release rate considerably due to the large volume of distribution in the average human patient [19]. The complexation efficiency (CE) value of 3.0 is unusually high; this suggests that three out of four SBECD molecules in solution are complexed with PTB molecules, which would also be an advantage for formulation, minimizing the amount of CD required in the dosage form [20].

## 4. Materials and Methods

### 4.1. Materials

Commercial pterostilbene (3,5-dimethoxy-4′-hydroxystilbene, PTB) used for the preparation of the binary systems was purchased from Candlewood Stars Inc. (Danbury, CT, USA). The cyclodextrins used, namely, hydroxypropyl-β-CD (HPβCD), heptakis(2,6-di-*O*-methyl)-β-CD (DIMEB), and randomly methylated β-CD (RAMEB) were generously provided by Cyclolab (Budapest, Hungary). All of the materials were sieved and the granulometric fractions with particle size <250 μm were collected. Sulfobutyl ether β-cyclodextrin (SBECD) sodium salt [(C_42_H_70-n_O_35_)(C_4_H_8_O_3_SNa)_n_, n = 14] was a gift from Cyclolab (Budapest, Hungary). All of the solvents used in the preparative methods were of analytical reagent grade.

The phosphate buffer saline solution (pH = 7.4) used in the phase solubility studies was prepared according to the European Pharmacopeia monograph [21].

### 4.2. Sample Preparation

Various methods were used to prepare the PTB inclusion complexes with the derivatized CDs. They include solution-phase techniques such as two different evaporation methods, semi-solid techniques such as the kneading method, and preparation via solid-based techniques, yielding simple physical mixtures and manually co-ground powders. The 1:1 TMB·PTB complex could be prepared by both kneading and the co-precipitation method.

#### 4.2.1. Physical Mixtures (PMs)

PMs were prepared by homogeneously mixing, in a Turbula, equimolar amounts of PTB with each CD. For the dimethylated derivative (DIMEB), an additional PM sample, namely, a PTB + DIMEB 1:2 (mol/mol) system, was prepared using an excess amount of the CD.

#### 4.2.2. Ground Products (GRs)

A small amount of each PM (≈30 mg) was subjected to manual trituration for 1 h using a mortar and pestle to promote the interaction.

#### 4.2.3. Kneaded Products (KNs)

About 50 mg of each PM was wetted with ethanol to obtain a doughy mass that was then dried in an oven at 70 °C to attain a constant weight. This procedure was repeated three times and then the samples were sieved through a 250-μm sieve.

#### 4.2.4. Microwave Products (MWs)

To obtain a clear solution, 50 mg of each PM was dissolved in 3 mL of a water–ethanol 8:2 (vol/vol) solution under magnetic stirring. Subsequently, the solution was evaporated using microwave irradiation at 425 W with a Pabisch CM-Aquatronic instrument (W. Pabisch, Milan, Italy) and the solid product obtained was passed through a 250-μm sieve to separate a homogeneous granulometric fraction.

#### 4.2.5. Rotavapor Products (RVs)

As an alternative drying method, the same hydroalcoholic solution used to isolate the MW products was evaporated using a rotary evaporator under reduced pressure. In this case, the solid obtained was also sieved to collect the particle size fraction <250 μm.

### 4.3. Methods

#### 4.3.1. Thermal Analysis

For the binary derivatized CD–PTB systems investigated, the temperature and enthalpy values were measured in triplicate using a Mettler STAR^e^ system (Mettler Toledo, Milan, Italy) equipped with a DSC821 Module and an Intracooler device for sub-ambient temperature analysis (Julabo FT 900, Julabo, Seelbach, Germany). The samples of 2–4 mg (Mettler M3 Microbalance) were placed in sealed aluminum pans with a pierced lid and analyzed in the temperature range 30–350 °C (heating rate β = 10 K min^−1^) with a nitrogen atmosphere (flow rate 50 mL min^−1^). The instrument was previously calibrated with indium as the standard reference.

A Mettler STAR^e^ TGA system (Mettler Toledo, Milan, Italy) with simultaneous DSC (TGA/DSC1) was used to measure mass losses upon heating the 5–8 mg samples in alumina crucibles with lids, with the same temperature range and N_2_-atmosphere used for the DSC above. The instrument was previously calibrated with indium as the standard reference.

Hot stage microscopy was performed on samples immersed in silicone oil (temperature range 30 to 350 °C; heating rate = 10 K min^−1^) under a Reichert (Arnsberg, Germany) polarized light microscope equipped with a Mettler FP82HT/FP80 system (Mettler Toledo, Novate Milanese, Italy). Photomicrographs were recorded at various time intervals during heating with a MOTICAM 2000 video camera (Motic, Milan, Italy).

For the TMB·PTB complex, thermogravimetric analysis (TGA) was performed on a TA-Q500 (New Castle, DE, USA) instrument (heating rate 10 K min^−1^, dry nitrogen gas flow rate 60 mL min^−1^) with samples (mass range 0.7–2.0 mg) placed in open pans. For DSC analysis, a TA Discovery DSC 25 instrument (New Castle, DE, USA) was used under analogous conditions to those for the TGA.

#### 4.3.2. Fourier Transform Infrared Spectroscopy

A Spectrum One FTIR spectrophotometer (64 scans at a resolution of 4 cm^−1^) (Perkin Elmer, Wellesley, MA, USA) equipped with a single reflection MIRacle^TM^ ATR accessory (Pike Technologies, Madison, WI, USA) was used for Fourier transform infrared (FTIR) mid-IR spectroscopy (650–4000 cm^−1^). The samples were pressed on an ATR crystal of ZnSe for the collection of the spectrum in transmittance mode, at least in triplicate.

#### 4.3.3. Phase Solubility Analysis

Phase solubility studies with HPβCD, DIMEB, and RAMEB were performed according to the method described by Higuchi and Connors [14]. The derivatized CDs were dissolved in phosphate buffered saline solution (PBS) to yield solutions whose concentrations were in the range 0–100 mM. An excess of PTB (about 200 mg) was added to 10 mL of each CD solution and the solutions were shaken for 48 h until equilibrium was reached, in a temperature-controlled water bath (T = 37 ± 1 °C). Aliquots were withdrawn, subsequently filtered through 0.22 μm nitrocellulose filters, and diluted appropriately with the same medium. The concentration of PTB was determined using UV–Vis spectrophotometry at a wavelength of 306 nm, using a Lambda 20 UV–Vis spectrophotometer (PerkinElmer, Milan, Italy). The extinction coefficient of PTB was calculated by dissolving 1 mg in 10 mL of an ethanol solution, as described in our previous work [8]. The solutions were protected from light to avoid PTB decomposition. The experiments were performed in triplicate.

For the phase solubility experiment with PTB and SBECD, five aqueous solutions of the CD with increasing concentrations (4, 8, 12, 16, and 20 mM) were prepared, the total volume of each solution being 4 mL. PTB was added in excess to each vial and the resulting suspensions were stirred at 250 rpm for 72 h at 23 °C with the vials protected from light. The unbuffered solutions were filtered with 0.2 μm nylon microfilters, further diluted to 1:100, and analyzed by high-performance liquid chromatography (HPLC) with a 60:40 (*v*/*v*) acetonitrile:water mixture as the mobile phase. A separate calibration curve for PTB was also constructed using HPLC following a similar procedure as that outlined by López-Nicolás et al. [18]. The flow rate, injection volume, column temperature, and detection volume were 0.6 mL/min, 5 μL, 23 °C, and 320 nm, respectively. A 5.20 mg sample of PTB was dissolved in 50 mL of a stock solution of acetonitrile:water (60:40 *v*/*v*) from which appropriate dilutions were made. Each diluted solution was analyzed at 23 °C by HPLC and the peak area for PTB was recorded. A calibration curve was constructed by plotting the peak area for PTB against the concentration of PTB. This was linear with an appropriate R^2^ value of 0.998 and the equation of the line was determined to be y = 10.914x − 57.83.

An Agilent 1220 Infinity LC system (Germany) consisting of a binary pump with sample degasser, an autosampler, a temperature-controlled column oven, and a UV-Vis variable wavelength detector. Separation was performed with an Agilent Poroshell 120 EC–C18 threaded column (4.6 mm × 50 mm × 2.7 µm) and the analytical data were recorded and analyzed using the program Agilent ChemStation [22].

#### 4.3.4. Job’s Method

Job’s method was used to determine the stoichiometries of the inclusion complexes between PTB and HPβCD, DIMEB and RAMEB. A total of 10 mL volumes of aqueous solutions containing different mole fractions of PTB (0 < X_PTB_ < 1) with each CD were prepared and maintained at 25 ± 1 °C. Job plots were obtained by plotting ΔA (the magnitude of the difference in the absorbance of PTB with and without CD) vs. the PTB mole fraction. The absorbance values were determined using UV–Vis spectrophotometry (λ_PTB_ = 306 nm).

The value of X_PTB_ at the maximum point on the curve corresponded to the stoichiometries of the inclusion complexes.

#### 4.3.5. ^1^H NMR Spectroscopy

^1^H NMR spectroscopy was used to determine the stoichiometry of the co-precipitated crystals of TMB·PTB. A 3 mg sample was dissolved in deuterated dimethyl sulfoxide (DMSO-d_6_) at 23 °C in a clean tube and the spectrum was recorded on a Bruker Ultrashield 400 Plus Spectrometer (Billerica, MA, USA). The program MestReNova [23] was used to analyze the resulting data.

#### 4.3.6. X-ray Diffraction

The PXRD patterns of the TMB·PTB complex (samples from the kneading and co-precipitated crystals) were recorded on a Bruker D2 Phaser desktop powder diffractometer (Billerica, MA, USA) with CuKα_1_ radiation (λ = 1.5406 Å) with the X-ray generator set at 30 kV and 10 mA. Samples were lightly ground to minimize the effects of preferred orientation and placed on a silicon zero-background sample holder. The scanning range was 4–40° 2θ with a step size of 0.0164° and a primary beam path slit of 0.6 mm. The unit cell dimensions were recorded on a Bruker D8 VENTURE single-crystal X-ray diffractometer (Madison, WI, USA) with graphite-monochromated MoKα radiation (λ = 0.71073 Å).

## 5. Conclusions

Relatively few reports have focused on fundamental issues such as the thermodynamics of the CD complexation of PTB and the determination of complex association constants, a notable exception being the seminal paper by López-Nicolás et al. [18]. In our recent paper [8], we reported the attempted preparation of solid inclusion complexes between the crystalline native CDs (α-, β-, and γ-CD) and PTB in both the solid state and in an aqueous solution as well as their characterization by a variety of physicochemical methods. Instead, the present study focused on determining the utility of a series of pharmaceutically more relevant, amorphous β-CD derivatives, namely, HPβCD, DIMEB, RAMEB, and SBECD, as potential host compounds for complexation with PTB in the solid-state, and in an aqueous solution as solubilizers for PTB. This study resulted in the isolation of new solid inclusion complexes of PTB and has clearly demonstrated the vastly superior PTB solubility enhancements achievable using these derivatized CDs, thereby contributing to the knowledge base of the supramolecular chemistry of PTB and the ongoing quest to render this compound more soluble for its development as a potential active pharmaceutical ingredient.

## Figures and Tables

**Table 1:** Thermal and enthalpic parameters from the DSC and TGA analyses recorded for the stable form I of PTB and the products obtained by the listed treatments.

Product	T_onset,melt_ (°C)	T_peak,melt_ (°C)	ΔH_melt_ (J g^−1^)	T_dec_ (°C) from TGA Analysis
Commercial	94.6 ± 0.1	95.4 ± 0.2	91 ± 1	195.8 ± 0.8
GR	93.7 ± 0.1	94.9 ± 0.1	89 ± 2	193.4 ± 0.2
MW	93.6 ± 0.1	95.1 ± 0.1	64 ± 3	189.5 ± 0.3
KN	94.2 ± 0.4	95.6 ± 0.6	94 ± 2	210.1 ± 0.1
RV	95.1 ± 0.4	95.8 ± 0.2	87 ± 4	190.4 ± 0.1

## Data Availability

Data is contained within the article and supplementary material.

## Supplementary Materials

The following supporting information can be downloaded at: https://www.mdpi.com/article/10.3390/ph16020247/s1, Figure S1: The structures of PTB and TMB (top) and the ^1^H NMR spectrum of the inclusion complex TMB·PTB. The sample was prepared by dissolving crystals of the complex obtained by co-precipitation in DMSO-d_6_; Figure S2: Calibration curve of the HPLC peak area for PTB versus the PTB concentration in mM (top) and the phase-solubility diagram for the SBECD–PTB system (bottom). The trendline corresponding to the equation is shown as a black curve.
