# Supplementary material for: Complexation between the Antioxidant Pterostilbene and Derivatized Cyclodextrins in the Solid State and in Aqueous Solution
Source: Pharmaceuticals (Basel). 2023 Feb 7;16(2):247. doi: 10.3390/ph16020247 (PMC9966925; doi:10.3390/ph16020247)
Supplement: Supplementary file 1 [file pharmaceuticals-16-00247-s001.zip › pharmaceuticals-2194234-supplementary.pdf]

SUPPLEMENTARY MATERIAL

Complexation between the antioxidant pterostilbene and derivatized cyclodextrins in the solid state and in aqueous solution

Laura Catenacci, Alexios I. Vicatos, Milena Sorrenti, Cesarina Edmonds-Smith, Maria Cristina Bonferoni and Mino R. Caira

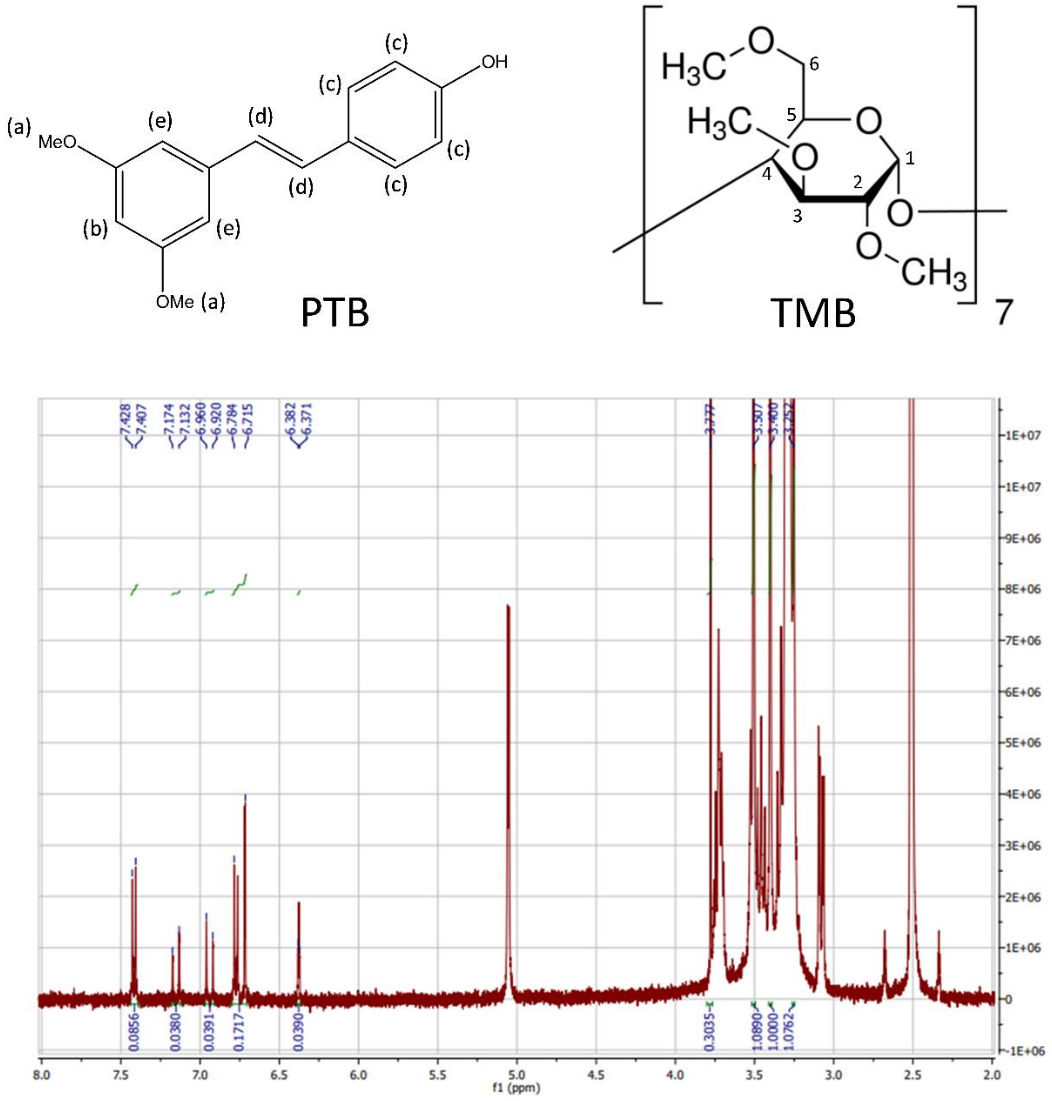

Figure S1. The structures of PTB and TMB (top) and the ^{1}H NMR spectrum of the inclusion complex TMB·PTB. The sample was prepared by dissolving crystals of the complex obtained by co-precipitation in DMSO-d_6.

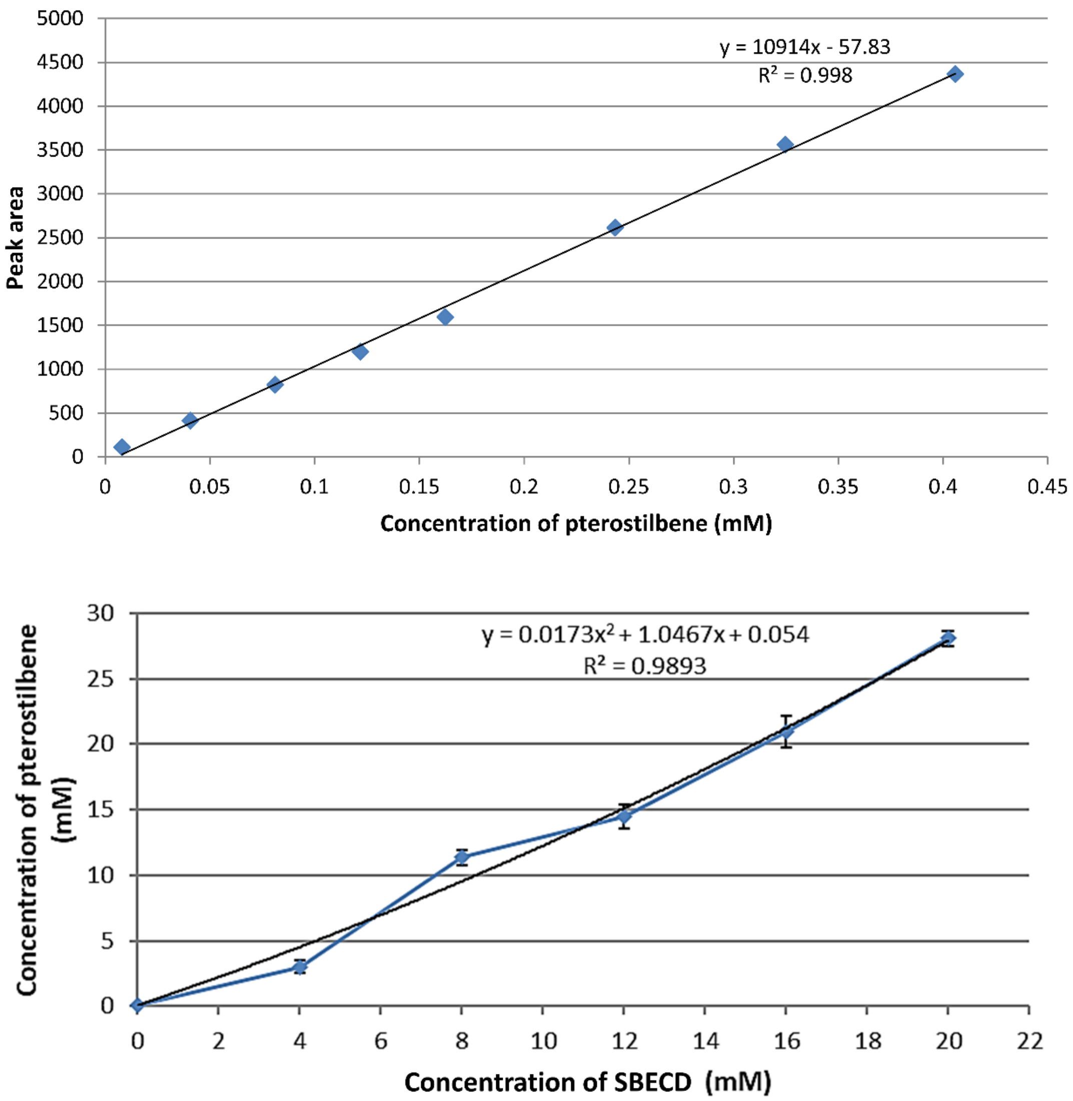

Figure S2. Calibration curve of the HPLC peak area for PTB versus PTB concentration in mM (top) and the phase-solubility diagram for the SBECD-PTB system (bottom). The trendline corresponding to the equation is shown as a black curve.
